# Effects of Light Color on the Growth, Feeding, Digestion, and Antioxidant Enzymes of *Tripneustes gratilla* (Linnaeus, 1758)

**DOI:** 10.3390/biology13020065

**Published:** 2024-01-23

**Authors:** Xinye Zhao, Yu Guo, Jiayang Li, Zhenhua Ma, Gang Yu, Chuanxin Qin

**Affiliations:** 1 South China Sea Fisheries Research Institute, Chinese Academy of Fishery Sciences, Guangzhou 510380, China; zhao13803362411@163.com (X.Z.); guoyu25895177@163.com (Y.G.); jiayangli0319@hotmail.com (J.L.); zhenhua.ma@hotmail.com (Z.M.); gyu0928@163.com (G.Y.); 2 Key Laboratory of Efficient Utilization and Processing of Marine Fishery Resources of Hainan Province, Sanya Tropical Fisheries Research Institute, Sanya 572018, China; 3 College of Marine Sciences, Shanghai Ocean University, Shanghai 201306, China; 4 Hainan Yazhou Bay Seed Laboratory, Sanya 572025, China

**Keywords:** sea urchin, LED light color, consume, growth, enzyme activity

## Abstract

**Simple Summary:**

Differing light color environments can have inhibitory and promotional effects on the growth of aquatic life. We aimed to determine the optimal light color composition that promotes the growth and development of *Tripneustes gratilla* and provide valuable basic insights for the large-scale cultivation of sea urchins in China. The sea urchins exhibited improved growth and feeding performance under blue and green light conditions, and the sea urchin organisms were in better physiological condition under blue light. In contrast, red light had a significant inhibitory effect on the feeding and growth rates of the sea urchins. These differences may differentially affect the development and reproduction of sea urchins, with sea urchins being more suited to growth and development under blue light, followed by white and green light, and not suited to development and reproduction under red light. Studying the impact of light color on the digestive and antioxidant processes of sea urchins is essential to decrease physiological stress and enhance the well-being of farmed sea urchins.

**Abstract:**

To study the effects of light color on sea urchin (*Tripneustes gratilla*), blue light (B, λ_450nm_), yellow light (Y, λ_585–590nm_), red light (R, λ_640nm_), green light (G, λ_510nm_), white light (W, λ_400–780nm_), and darkness (H) groups were established in a recirculating seawater aquaculture system. Six different LED light color treatment groups with a photoperiod of 12 L:12 D were tested for 30 days to investigate the effects of different light colors on the feeding, growth, and enzyme activities of *T. gratilla* (142.45 ± 4.36 g). We found that using different LED light colors caused significantly different impacts on the feeding, growth, and enzyme activity of *T. gratilla*. Notably, the sea urchins in group B exhibited better growth, with a weight gain rate of 39.26%, while those in group R demonstrated poorer growth, with a weight gain rate of only 26%. The feeding status differed significantly (*p* < 0.05) between groups B and R, with group B consuming the highest daily intake (6.03 ± 1.69 g) and group R consuming the lowest (4.54 ± 1.26 g). Throughout the three phases, there was no significant change in the viability of the α-amylase (*p* > 0.05). Conversely, the pepsin viability significantly increased (*p* < 0.05) in group B. The lipase viability consistently remained at the lowest level, with no notable differences between group W and group B. In group R, both the α-amylase and pepsin viabilities remained lower, whereas the lipase viability was noticeably greater in each phase than in group B (*p* < 0.05). Among the antioxidant enzymes, group R exhibited a trend of initial increase followed by decreases in catalase, superoxide dismutase, and glutathione peroxidase activities, particularly during the third stage (15–30 days), during which a significant decrease in antioxidant enzyme activity was observed (*p* < 0.05). Taken together, these findings suggest that blue light positively affects the growth, feeding, digestion, and antioxidant capacity of *T. gratilla* in comparison with those in other light environments, whereas red light had an inhibitory effect. Furthermore, *T. gratilla* is a benthic organism that lives on shallow sandy sea beds. Thus, as short wavelengths of blue and green light are more widely distributed on the seafloor, and long wavelengths of red light are more severely attenuated on the seafloor, shorter wavelengths of light promote the growth of bait organisms of sea urchins, which provide better habitats for *T. gratilla*.

## 1. Introduction

Sea urchins can evolve adaptive survival mechanisms over time in response to their environment. Environmental factors, such as light, are essential for the biological growth, development, reproduction, and behavior of marine organisms. The color, intensity, and photoperiod of light can crucially impact these factors [1]. Light absorption and penetration in waters of different qualities are well known, and the absorption of light color in water is correlated with the wavelength and water depth [2,3,4]. Furthermore, differing light color environments can have inhibitory or promotional effects on the growth of aquatic life [5]. Domestic and international scholars verified that the color of light impacts the growth and development of organisms. When *Cyprinus carpio* is raised under red light, its feed conversion rate, growth performance, and survival rate significantly increase [6]. Blue and green light promote the endocrine secretion of *Verasper moseri*, which has a relatively high growth rate [7], and green light increases the hatchability and production of *Haliotis discus hannai*, which increases commercial benefits [8]. Yang et al. [3,9] reported that short wavelengths (blue light, green light) promote the growth and foraging of intermediate globe sea urchins and that red light inhibits the growth of sea urchins; however, the foraging of sea urchins exposed to blue light for a long period gradually decreases. Therefore, the effects of different light colors on aquatic organisms are clearly species specific.

When aquatic organisms experience a stress response due to changes in the external environment, their various enzyme activities are altered, thus affecting their normal physiological functions and activities [10]. The investigation of digestive enzymes in the intestinal tract of organisms is crucial for comprehending the physiological functions of sea urchins. This approach can offer insights into the fundamental characteristics of the digestive physiology in aquatic animals [11]. For example, the digestive enzyme activities of *Oreochromis niloticus* significantly increase under red- and white-light conditions [12], whereas the trypsin activity of *Lateolabrax maculatus* significantly decreases under red light [13]. Organisms respond to environmental changes and possess a well-developed antioxidant system to maintain free radical balance and reduce oxidative damage [14]. However, the effect of light color on mollusks may be different from that on fish. In a blue-light environment, the succinate dehydrogenase activity of *Haliotis discus hannai* was significantly greater than that in the red- and white-light groups, whereas the lactate dehydrogenase activity was significantly lower than that in the red- and white-light groups [8]. The results demonstrated that light color substantially impacts the digestive and antioxidant enzyme activities in different categories of aquatic organisms, highlighting the crucial regulatory role of light in physiological metabolism [2]. Recent studies on sea urchin digestive and antioxidant enzymes have focused on assessing the effects of bait [15] and environmental factors [16,17] (salinity, pH, temperature), and less research has been carried out on the effects of light color on sea urchin digestive and antioxidant enzymes.

Light color is a crucial factor in shallow waters and affects the feeding, growth, and development of sea urchins [18]. *Tripneustes gratilla* is among the few edible sea urchins that exist in tropical regions [19]. They inhabit sandy-bottom areas in shallow waters where seagrasses are abundant and feed on macroalgae and seagrasses [20,21,22] and are widely distributed in the shallow waters of Hainan, Taiwan, and Sansha, China [23]. In this study, *T. gratilla* was selected as the research subject because of its excellent characteristics, such as full gonads [24], closed life cycle [25], high growth rate [26], and high market value [24]. Recent studies on *T. gratilla* have focused on the effects of different baits on the growth and reproduction of sea urchins [27,28,29], the feeding preferences of sea urchins for algae [24,30], and the population growth patterns and distribution of sea urchins [31,32]. *T. gratitlla* is a benthic organism that lives on a shallow sandy seabed where short-wavelength blue and green light is more widely distributed and long-wavelength red light is more severely attenuated. However, studies on the effects of light color on the growth and development and digestive antioxidant physiological mechanisms of *T. gratilla* are rare, and how light color affects its growth and development remains unclear.

In this study, we designed five light and dark groups to analyze the effects of light on the growth, development, feeding, digestion, and antioxidant activity of *T. gratilla* in an indoor aquaculture facility. Our objective was to determine the optimal light color composition for promoting the growth and development of *T. gratilla* and to provide fundamental theories for the large-scale cultivation of these organisms in China. Studying the impact of light color on the digestive and antioxidant processes of *T. gratilla* is essential for decreasing physiological stress and enhancing the well-being of farmed sea urchins.

## 2. Materials and Methods

### 2.1. Sea Urchins

This study was executed during April and May 2023 at the Tropical Development Centre of the South China Sea Fisheries Research Institute of the Chinese Academy of Fisheries Sciences. The sea urchins utilized for the study were procured from the sea vicinity close to Xincun town, Lingshui Lizu Autonomous County, Hainan Province. Using HOBO, the light intensity at the submerged sea urchins was 179 lux, the water depth was 6.2 m, and the transparency was 4.0 m. The urchins were domesticated in a bucket for 7 days before the start of the experiment. The seawater temperature was 29.5 ± 0.72 °C; the salinity was 31.4 ± 0.86; the pH was 8.11 ± 0.24; and the urchins were subjected to a natural light cycle, adequate oxygen dissolution, fresh seaweed feeding during the staging period, daily bottom suction, and 1 water change every 2 days.

### 2.2. Experimental Design

Six experimental groups were established: blue light (B, λ_450nm_, 201 lux), yellow light (Y, λ_585–590nm_, 202 lux), red light (R, λ_640nm_, 198 lux), green light (G, λ_510nm_, 199 lux), white light (W, λ_400–780nm_, 206 lux), and dark (H) groups. The light source was 10 W, the light source was 15 cm from the water surface, and the photoperiod was 12 L:12D.

We randomly selected 168 healthy sea urchins and measured their body weight; the average test diameter was 82.27 ± 4.36 mm, the average test height was 55.44 ± 5.47 mm, and the average wet body weight was 142.45 ± 4.36 g. The sea urchins were randomly divided into six groups (three parallels in each group) and placed into a recirculating aquaculture tank (length 60 × width 60 × height 40 cm), with nine sea urchins in each tank. The tanks were fully oxygenated, with a temperature of 29.5 ± 0.43 °C, salinity of 31.2 ± 0.92, and pH of 8.11 ± 0.38. The urchins in each group were cultivated for 30 days under the same cultivation conditions.

### 2.3. Sample Collection

Samples were collected on the 7th, 15th, and 30th days of the test, and three sea urchins with good viability were randomly selected from each group. The body size of each sea urchin was measured, and the urchins were placed on an ice box for vivisection. Approximately 2 mL of body cavity fluid was extracted from each sea urchin and placed in a centrifuge tube, after which the supernatant was centrifuged (3500 rpm) for 10 min and subsequently transferred to a new centrifuge tube. Immediately after extraction of the luminal fluid, the gut tissue was clipped and frozen in liquid nitrogen, and the same centrifuged luminal fluid was stored at −80 °C.

### 2.4. Measurement and Analysis of Samples

#### 2.4.1. Growth Performance Measurement

The weight growth rate and specific growth rate were calculated as follows:

(1) $$WGR=\frac{W_{f}-W_{i}}{W_{i}}\times 100\%\;SGR=In\frac{X_{f}}{X_{i}}/T\times 100\%$$

where *WGR* is the weight increase rate (%) of the sea urchin, *W_i_* is the initial body weight (g) of each urchin, *W_f_* is the final body weight (g) of each urchin, *SGR* is the specific growth rate (%) of the sea urchin, *X_f_* is the final value of each stage of the trait (test diet and weight), *X_i_* is the initial value of the trait, and *T* is the duration of the stage (d).

#### 2.4.2. Feeding Performance Measurement

During the experimental period, kelp was provided every 2 days. The mass of the dried kelp (*W*_0_) was weighed before each feeding, and after 48 h, the unfed kelp was removed and dried at 40 °C, after which the weight of the remaining kelp (*W*_1_) was determined.

The daily food intake and daily food intake rate were calculated as follows:

(2) $$R=W_{0}-W_{1}/\left(N\cdot A\right)\;F=R/W_{m}$$

where *R* is the daily intake (g·(pcs·d)^−1^) of the sea urchins, *N* is the number of sea urchins (pcs), *A* is the number of feeding days (d), *F* is the daily intake rate (%) of *T. gratilla*, and *W_m_* is the average body mass of sea urchins (g).

#### 2.4.3. Enzyme Activity Measurement

At different sampling times, the intestinal tissues of three rows of white spiny sea urchins were collected, and the samples were ground with 0.2 mol·L^−1^ saline at a ratio of 1:9 by mass volume. The ground solution was centrifuged at 4 °C and 5000 rpm for 10 min, and 1 mL of the supernatant was collected in a clean EP tube and placed at 80 °C until analysis. The enzyme activities were determined using the appropriate kits (Nanjing Jiancheng Bioengineering Institute); the activities of α-amylase and lipase were measured in the intestinal tract; and the activities of superoxide dismutase (SOD), glutathione peroxidase (GSH-PX), and catalase (CAT) were measured in the body cavity supernatant, with three parallels in each group.

### 2.5. Data Analysis

Excel 2019 was used to process the data, Origin 2022 was used for graphing, and SPSS 27.0 software was used to statistically analyze the experimental data. The Kolmogorov–Smirnov test and Levene’s test were used to analyze the normality and chi-square of variance. One-way ANOVA was used to determine whether there was a significant difference between the treatment groups, and the significance of the differences between the means (*p* < 0.05 indicates that the differences were significant, *p* > 0.05 indicates a nonsignificant difference) was determined using Duncan’s method.

## 3. Results

### 3.1. Growth Performance of Sea Urchins under Different Light Colors

**Figure 1:**
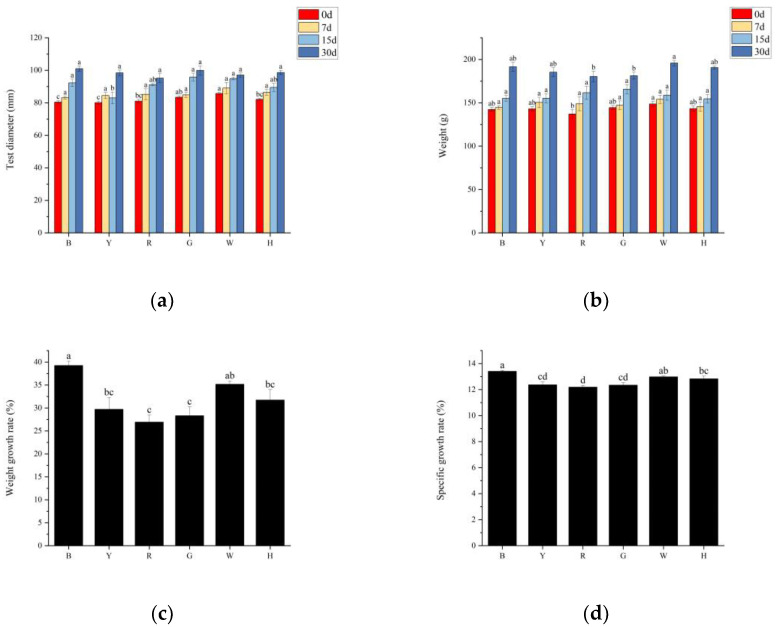
Test diameter (**a**) and weight gain (**b**) of *T. gratitlla* at different times under six light color treatments, as well as the rate of sea urchin weight increase (**c**) and specific growth rate (**d**) throughout the experiment. The six color groups were blue light (B), yellow light (Y), red light (R), green light (G), white light (W), and dark (H). Different letters indicate significant differences between the six color groups (*p* < 0.05).

Different light colors significantly affected the growth of *T. gratilla*, and the test diameters and body weights of the sea urchins in all groups gradually increased, with significant differences between the initial and final body weights (*p* < 0.05); the highest weight gain of sea urchins in group B was 49.21 ± 2.31 g. In the first stage (0~7 days), the body weights of the different groups did not significantly differ (*p* > 0.05), but the test diameters and weights of groups W and H were significantly greater than those of the other groups (*p* < 0.05); in the second stage (7~15 days) and third stage (15–30 days), there was no significant difference (*p* > 0.05) in the shell diameters or body weights between the groups (Figure 1a,b). The trends in the weight gain rates (WGRs) and specific growth rates (SGRs) were consistent between the groups with different light color treatments. The WGR and SGR of the sea urchins in group B were the highest (39.26% and 13.41%, respectively), which were significantly greater than those in the other groups (*p* < 0.05), and those of the sea urchins in group R were the lowest (26.93% and 12.19%, respectively), which were significantly lower than those in groups B and W (*p* < 0.05) (Figure 1c,d). No sea urchins died during the experiment.

### 3.2. Feeding Performance of Sea Urchins under Different Light Colors

**Figure 2:**
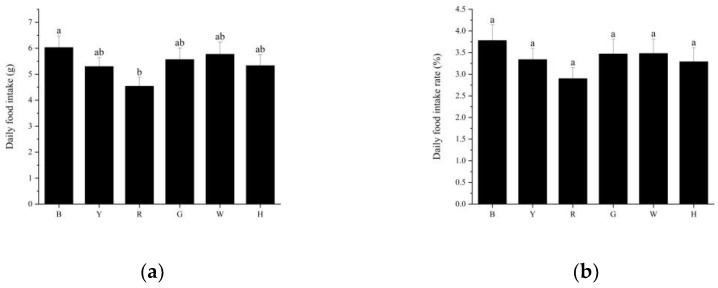
Daily food intake (**a**) and daily food intake rate (**b**) of *T. gratitlla* under different spectral treatments. Different letters indicate significant differences between the six color groups (*p* < 0.05).

The trends in daily food intake and daily feeding rate per sea urchin were consistent between the different treatment groups (Figure 2a,b), with group B (6.03 ± 1.69 g) consuming the most food, followed by groups W (5.77 ± 1.78 g), G (5.57 ± 1.65 g), H (5.33 ± 1.59 g), Y (5.30 ± 1.30 g), and R (4.54 ± 1.26 g). There was a significant difference in the daily food intake of sea urchins between groups B and R (*p* < 0.05). There was no significant difference (*p* > 0.05) in the daily intake of sea urchins or the daily intake rate between the remaining groups.

### 3.3. Changes in the Digestive Enzyme Activities of Sea Urchins under Different Light Colors

**Figure 3:**
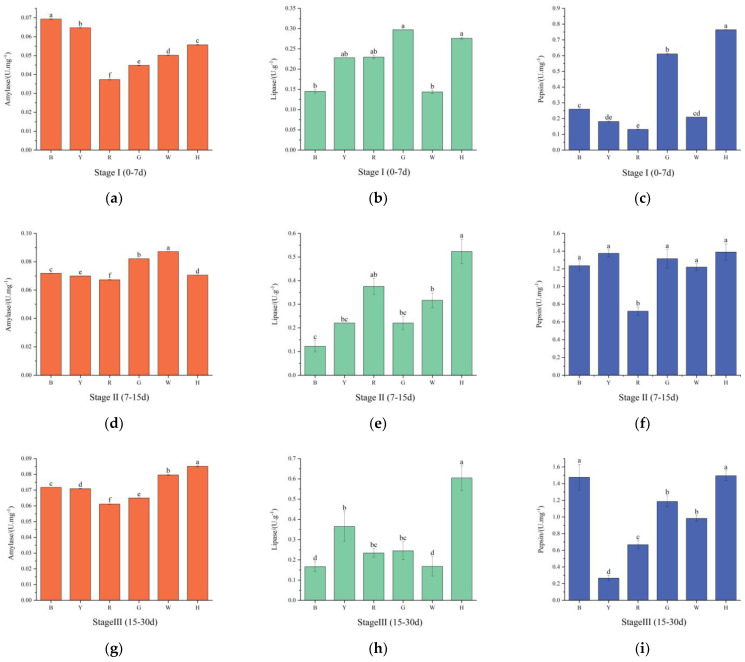
Changes in the amylase (**a**,**d**,**g**), lipase (**b**,**e,h**) and pepsin (**c**,**f**,**i**) activities of sea urchins at various stages under different light color treatments. Different letters indicate significant differences between the six color groups (*p* < 0.05).

As shown in Figure 3a,d,g, the AMS activities in groups B and Y were stable with increasing test time, while the AMS activity in the remaining groups tended to increase and then decrease. In the first stage, there were significant differences in the AMS activities between the groups (*p* < 0.05), with group B having the highest activity, followed by groups Y, H, W, G, and R. In the second stage, the AMS activities increased in groups H, W, G, and R compared with the first stage, and there were significant differences (*p* < 0.05) between the groups, with group W having the highest activity and group R having the lowest activity. In the third stage, the AMS activity decreased compared with that in the second stage, and the difference was significant (*p* < 0.05); the highest activity was found in group H, and the lowest activity was found in group R.

As shown in Figure 3b,e,h, in the first stage, the LPS activities of group Y and group R were not significantly different from those of the other groups (*p* > 0.05); group G had the highest LPS activity, and group B had the lowest activity. In the second stage, compared with those in the first stage, the LPS activities in the R, H, and W groups increased; the activity in the Y group was stable; and the activities in the other groups decreased. In the third stage, the LPS viabilities increased in all groups except for groups R and W. Group H had the highest viability, and there was a significant difference in the LPS viability between this group and the other groups (*p* < 0.05).

As shown in Figure 3c,f,i, with increasing test time, the pepsin activity of group R increased and then stabilized, the pepsin activities of groups B and H gradually increased, and the pepsin activities of the remaining groups tended to increase and then decrease. In the first phase, the pepsin activities were significantly greater in groups H and G than in the other groups (*p* < 0.05). In the second stage, the pepsin activities increased in all groups compared with those in the first stage, with the lowest activity occurring in group R, which significantly differed from those in the other groups (*p* < 0.05). In the third stage, the pepsin activities increased in groups B and H, which significantly differed from those in the other groups (*p* < 0.05), and group Y had the lowest pepsin activity.

### 3.4. Changes in the Antioxidant Enzyme Activities of Sea Urchins under Different Light Colors

**Figure 4:**
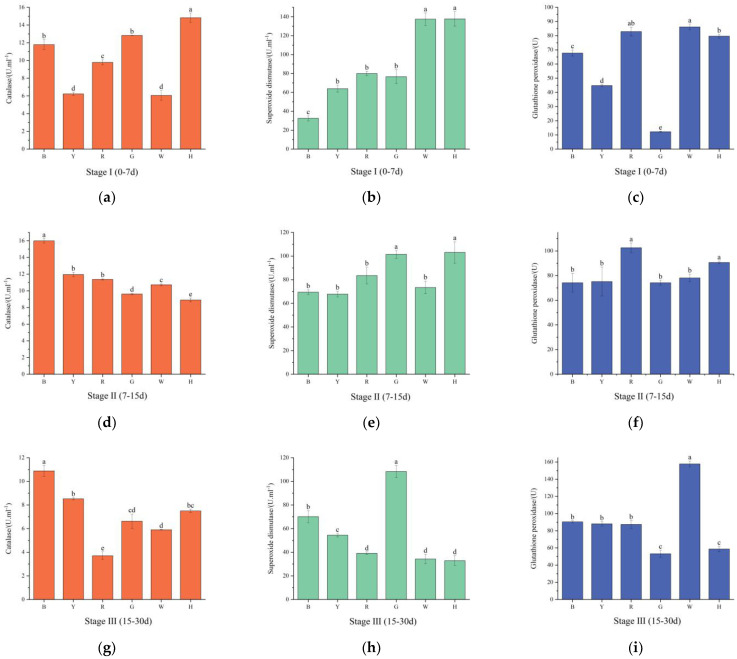
Changes in the catalase (**a**,**d**,**g**), superoxide dismutase (**b**,**e**,**h**) and glutathione peroxidase (**c**,**f**,**i**) activities of sea urchins at various stages under different light color treatments. Different letters indicate significant differences between the six color groups (*p* < 0.05).

As shown in Figure 4a,d,g, with increasing test time, the CAT activity was the highest in group H in the first phase, which significantly differed from those in the remaining groups (*p* < 0.05). In the second stage, the CAT activities decreased in groups G and H and increased in the remaining groups, with the lowest CAT activity occurring in group H, which significantly differed from those in the remaining groups (*p* < 0.05). In the third stage, the CAT activities decreased in all groups, and the highest activity was found in group B, while the lowest activity was found in group R. There were significant differences (*p* < 0.05) between the groups.

As shown in Figure 4b,e,h, the SOD activities gradually decreased in groups H and W with increasing test time, while they increased and then decreased in the remaining groups. The highest SOD activity was observed in group H, and the lowest activity was observed in group B in the first stage; these two groups significantly differed from the other groups (*p* < 0.05). In the second stage, the differences in SOD activities between groups G and H and between the other groups were significant (*p* < 0.05), and the highest SOD activity was found in group H. In the third stage, the SOD activity in group G was significantly greater than those in all the other groups (*p* < 0.05).

As shown in Figure 4c,f,i, the GSH-PX activities increased and then decreased in groups R, G, and H. The GSH-PX activity decreased and then increased in group W. The activity gradually increased in the other groups. In the first stage, the expression in group G was significantly lower than those in the other groups, and there were significant differences between the groups (*p* < 0.05). The GSH-PX activity was the highest in group R in the second stage, and there was a significant difference between this group and the other groups (*p* < 0.05). The GSH-PX activity was significantly increased in group W in the third stage (*p* < 0.05).

## 4. Discussion

### 4.1. The Effect of Light Color on the Feeding and Growth of T. gratilla

We hypothesized that the positive effect of blue light on sea urchin feeding was due to the short-wavelength light color, which was similar to the light color composition of sea urchins in their natural habitat and that sea urchins could better adapt to such light conditions, which could lead to better feeding and promote individual growth and development. In the present study, *T. gratilla* exhibited a greater feeding rate under blue light. There were some differences in the orders of the weight gain rates and feeding rates between groups G, Y, and H, which could have been caused by the different bait conversion rates of sea urchins after feeding under different light colors. The results suggested that blue light (a short-wavelength light) is more conducive to sea urchin feeding, while red light (a long-wavelength light) may increase the adaptive stress of sea urchins in the environment. We believe that due to the high density of phytoplankton and dissolved organic matter [33], light (especially blue light) has a reduced ability to be projected in intertidal and shallow subtidal waters, thus increasing the range of distribution of long wavelength light (red and yellow light) [18,34].

Macroalgae, as bait sources for sea urchins, directly affect the growth of sea urchins [9], and light color is one of the important factors for the growth and distribution of macroalgae [35,36,37]. Algae absorb wavelengths of light differently, such as brown algae, which use more blue-green light [38], and blue light can enhance the growth and reproduction of *Saccharina japonica* [39,40]. Scott Seymour [24] reported that *T. gratilla* prefers to feed on brown algae; therefore, we suggest that blue light can promote the growth of brown algae in the field, thus providing more food for *T. gratilla*. Furthermore, blue light not only promotes sea urchin feeding but is also important for macroalgal growth, whereas sea urchins under red light have less bait and poorer feeding performance. Light color may affect not only the feeding and prey sources of sea urchins but also the feed conversion rate, and further studies of these phenomena based on energy metabolism are needed.

The effects of light color composition on marine organisms vary according to the species and their growth and development, and the SGR and WGR are important reference indices for evaluating the growth of organisms and are the most intuitive indicators [41]. For example, *Dicentrarchus labrax* exhibited an increased SGR and WGR under red light [42], and Korean rockfish exhibited an improved growth and feeding performance under green, blue, and white light [2]. In this study, all growth parameters of the sea urchins in the B light group were significantly greater than those in the other treatment groups, and the greatest increase in growth weight (55.89 ± 2.32 g) was observed in the B group, followed by those in the W, H, Y, and G groups, while the lowest increase in growth weight (41.06 ± 4.94 g) was observed in the R group. Taken together, these results indicated that the overall growth of *T. gratilla*, as measured by various growth indices, was good in the blue light environment, in contrast with those of the sea urchins under red light, which had the lowest weight gain and specific growth rate and were significantly lower than those of the other groups (*p* < 0.05).

Green light has a short wavelength, and the growth of white spiny three-row sea urchins was slower in this experiment, probably due to the larger initial sea urchin size and the faster growth of small sea urchins [43]. However, the higher WGR in the white group with a greater initial size of sea urchins was considered because the white light was closer to the growth environment of *T. gratilla* during transient incubation, thus reducing the aquaculture process for better development and growth.

### 4.2. The Effect of Light Color on the Digestive Enzyme Activities of T. gratilla

Digestive enzyme activity is an important indicator of the digestive capacity of aquatic organisms and can directly reflect the development and nutritional status of the digestive tracts of organisms [44]. Changes in light color cause the body to lose more energy and digestive enzymes are required to be more active in digesting food to ensure proper energy supply [45]. Currently, studies on the effect of light color on the digestive enzyme activities of sea urchins are rare. Chen Jisheng [46] reported that optimal digestive enzyme activities could be maintained in *Anthocidaris crassispina* planktonic larvae under 500 lux light intensity. Shi Jiageng [47] investigated the effects of the mixing ratios of different algae, namely, *Leathesia difformis* and *Ulva pertusa*, on the digestive enzyme activities of sea urchins; the biodigestibility of *U. porea* was better than that of *L. difformis*. The results showed that in the three stages, the change in α-amylase activity in the B group was not significant (*p* > 0.05), the increase in pepsin activity was significant (*p* < 0.05), the increase in lipase activity gradually decreased, and the changes in the W group were similar to those in the B group. The α-amylase and pepsin activities of the R group continued to be at a lower level, and the lipase activity was significantly greater than that of the B group in all three stages (*p* < 0.05).

The lipase level is an important indicator of the maturity and function of the digestive system of sea urchins [48]. The decrease in LPS viability in group B in the second stage may have been due to the faster growth of sea urchins under blue light, which inhibited the LPS activity in sea urchins and led to a decrease in viability. The greater viability of lipase in group R may have been because the sea urchins in the red group environment could use fat [49] to counteract the effects of environmental stress. Under blue light, sea urchins are less stressed by external environmental changes and have higher overall digestive enzyme activity, which enhances their digestive ability, and thus, promotes feeding and growth. In addition, a high proportion of protein and starch in bait increases the viability of echinoderms harboring α-amylase and pepsins [48]. The mechanism by which bait composition affects the digestive enzymes of sea urchins under different light colors needs further investigation.

### 4.3. The Effect of Light Color on the Antioxidant Enzyme Activities of T. gratilla

The antioxidant system of aquatic animals removes reactive oxygen species generated during metabolism from the organism in a timely manner, and under environmental stress, many reactive oxygen species are generated in the organism, resulting in organismal damage [50]. GSH, which is a tripeptide compound containing sulfhydryl groups, has strong reducing properties and is important for maintaining organismal homeostasis and preventing oxidative stress, whereas CAT can decompose H_2_O_2_ produced by SOD after the conversion of reactive oxygen species, thus reducing its toxicity. CAT, SOD, and GSH-PX can effectively scavenge oxygen radicals, reduce oxidative damage in the body, and increase the body’s immunity [51,52].

In the present study, it was found that the antioxidant enzyme activities of sea urchins subjected to different light colors changed with increasing test time in the body cavity fluid. In all three stages, the CAT, SOD, and GSH-PX activities in group R tended to increase and then decrease. The excessive generation of free radicals in cells and tissues can lead to oxidative stress when the antioxidant defense system is not able to counteract their effects [53]. It was hypothesized that *T. gratilla* in group R exhibited a greater level of oxidative stress during the first phase. When the accumulation of reactive oxygen species in an organism exceeds the scavenging capacity of antioxidant enzymes, excess toxic free radicals in the body can inhibit antioxidant enzyme activity [54,55]. In the third stage, the activities of CAT, SOD, and GSH-PX in the R group decreased significantly (*p* < 0.05), which was presumed to be because red light had already caused damage to the sea urchin and because the antioxidant capacity had weakened [17]. The nonsignificant (*p* > 0.05) changes in antioxidant enzyme activity in the blue light group at all stages may have been because sea urchins are better adapted to blue light, and the free radical content in the organism changes less.

## 5. Conclusions

In summary, different light colors had different effects on the growth, feeding, and digestive metabolism of *T. gratilla* under a 12 L:12 D cycle and 200 lux light intensity. The sea urchins exhibited improved growth and feeding performance under blue and green light conditions, and the sea urchin organisms exhibited better physiological conditions under blue light. In contrast, red light had a significant inhibitory effect on the feeding and growth rates of sea urchins. Therefore, the results suggested that *T. gratilla* was most suited to growth and development under blue light, followed by white and green light, and not suited to development and reproduction under red light. However, studies investigating the effects of light color on the growth and development of *T. gratilla* are limited, and the exact physiological mechanisms, including the effects of different sizes and larval development, are not fully understood. Additional studies investigating the effects of different light colors on sea urchin larval growth and development are needed to provide a theoretical basis for a more selective and suitable environment for *T. gratilla* reproduction.

## Data Availability

Data are available upon request to the corresponding author.
